# Mechanically aligned total knee arthroplasty with the extension-first technique does not equally restore neutral knee alignment in all preoperative knee phenotypes

**DOI:** 10.1007/s00167-022-07147-4

**Published:** 2022-09-10

**Authors:** Nina Hörlesberger, Carina Zinggl, Maria Anna Smolle, Lukas Leitner, Birgit Lohberger, Andreas Leithner, Patrick Sadoghi

**Affiliations:** Department of Orthopaedics and Trauma, Medical University of Graz, Auenbruggerplatz 5, 8036 Graz, Austria

**Keywords:** Knee prosthesis, Radiography, Arthroplasty, Replacement, Knee/instrumentation, Biomechanical phenomenon, Arthroplasty, Replacement, Knee/methods, Mechanical alignment, Leg axis, Knee/phenotypes

## Abstract

**Purpose:**

The aim of this study was to determine the change in the long leg axis according to the preoperative knee phenotype using the mechanically aligned extension-first technique in total knee arthroplasty. The hypothesis of this study was that the knee phenotype would have an impact on the postoperative leg axis.

**Methods:**

This was a retrospective comparative study comprising 224 whole-leg radiographs of 112 patients. The leg axes of the pre- and postoperative radiographs were measured and categorized into three preoperative limb phenotypes (based on the hip-knee-ankle angle [HKA]) according to Hirschmann et al. (varus—HKA < 178.5°, neutral—HKA 178.5°–181.5°, and valgus—HKA > 181.5°). Additionally, femoral phenotypes (based on the femoral mechanical angle [FMA], i.e., the mechanical medial distal femoral angle [mMDFA], as well as the tibial phenotypes [based on the tibial mechanical angle, i.e., the medial proximal tibial angle (MPTA)] was calculated. The change in the long leg axis was analyzed and compared with the preoperative limb phenotype.

**Results:**

Significantly more patients with preoperative varus alignment shifted to neutral alignment (46.3%, *n* = 31) than did patients with preoperative valgus alignment (38.9%; *n* = 14). Moreover, 43.3% of patients (*n* = 29) with the varus phenotype remained in a varus alignment, compared with the 58.3% of patients with preoperative valgus phenotype (*n* = 21) remaining in valgus alignment. These findings were similar for both females (*p* < 0.001) and males (*p* = 0.015).

**Conclusion:**

Using an extension-first mechanically aligned surgical technique, varus phenotypes predominantly result in neutral leg axes or remain varus, neutral phenotypes remain neutral, and valgus phenotypes remain valgus or change to neutral phenotypes. This study showed that preoperative knee phenotypes in valgus knees influence this technique more strongly than estimated in previous investigations, which is in line with modern alignment philosophies for TKA.

**Level of evidence:**

Level IV, retrospective comparative study.

## Introduction

Neutral mechanical alignment has been established as the gold standard for total knee arthroplasty (TKA) for decades, providing good long-term survival and clinical outcomes. However, recent studies propose a residual varus alignment that is indicative of superior results [1, 10, 16, 20, 27, 28]. While Matziolis et al. found no significant difference in postoperative WOMAC (Western Ontario and McMaster Universities Arthritis Index) and Short Form-36 (SF-36) scores between patients with varus or neutral alignment [16], Vanlommel et al. reported that patients with postoperative mild varus achieved significantly higher WOMAC and KSS (Knee Society Score) scores [28].

In addition, mechanical or anatomical alignment techniques do not always result in a neutral leg axis [3, 12] and are under criticism because of the abnormal kinematics and lack of reconstruction of the constitutional anatomy and laxity [22, 23]. A more advanced approach was the adjusted mechanical alignment technique, which left a residual varus based on the constitutional varus concept described by Bellemans et al. but revealed no clinically superior outcome [1].

Therefore, there has been a growing interest in more personalized approaches based on preexisting phenotypes called constitutional or functional alignment in TKA [7–9, 17]. The KA technique has been reported to show more personalized physiological implantation and superior clinical outcomes by restoring the anatomy and ligament balance of the constitutional leg axis before arthritic deformation, as proposed by Howell et al. [3, 21, 23].

However, no study has investigated the impact of the preoperative knee phenotype on radiological outcomes after mechanically aligned TKA with respect to this new knowledge. It is, therefore, of interest whether and to what amount conventional TKA techniques consider the preoperative limb phenotype, as there is controversy regarding which limb phenotype changes more using the extension-first, gap-balanced technique in TKA.

The aim of this study was therefore to determine the amount of change in the long leg axis depending on the preoperative knee phenotype using the mechanically aligned extension-first, gap-balanced technique. The hypothesis was that the preoperative limb phenotype would have an impact on the extent of change in the postoperative leg axis.

## Materials and methods

### Study design and recruitment

**Fig. 1:**
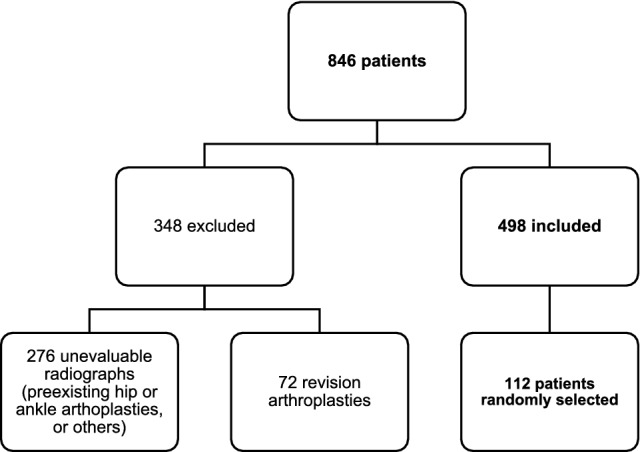
Flowchart of included and excluded patients who underwent primary total knee arthroplasty

This was a retrospective comparative level IV study of 224 radiographs including 112 patients. For recruitment, the hospital database was searched for patients who underwent implantation of TKA. The inclusion criterion was primary osteoarthritis. Exclusion criteria were preexisting arthroplasties of the hip or the ankle joint, revision arthroplasties, or secondary osteoarthritis. Patients who underwent total hip or ankle arthroplasty were excluded from the analysis (Fig. 1). All surgeries were performed by or under the supervision of the same senior surgeon (last author).

### Surgical technique, inpatient rehabilitation and radiological assessment

Upon admission, standardized radiographs of the knee joint in three planes (AP and lateral projection, and patella tangential projection) as well as full leg radiographs were taken. All prostheses (Attune DePuy Synthes, Warsaw, USA) were implanted under general or epidural anesthesia. A medial parapatellar approach was used, and surgery was performed under single-shot antibiosis with cefazolin or clindamycin. Knees were aligned using the extension-first technique, and rotation of the femoral shield was achieved using the gap-balanced technique [11]. All protheses were fully cemented, and patients were allowed to bear full weight postoperatively. Two days after surgery, continuous passive motion was performed until hospital discharge. Before discharge, standardized radiographs of the knee joint in three planes (AP and lateral projection, and patella tangential projection) as well as full leg radiographs were taken.

### Measurements of the radiographs

**Fig. 2:**
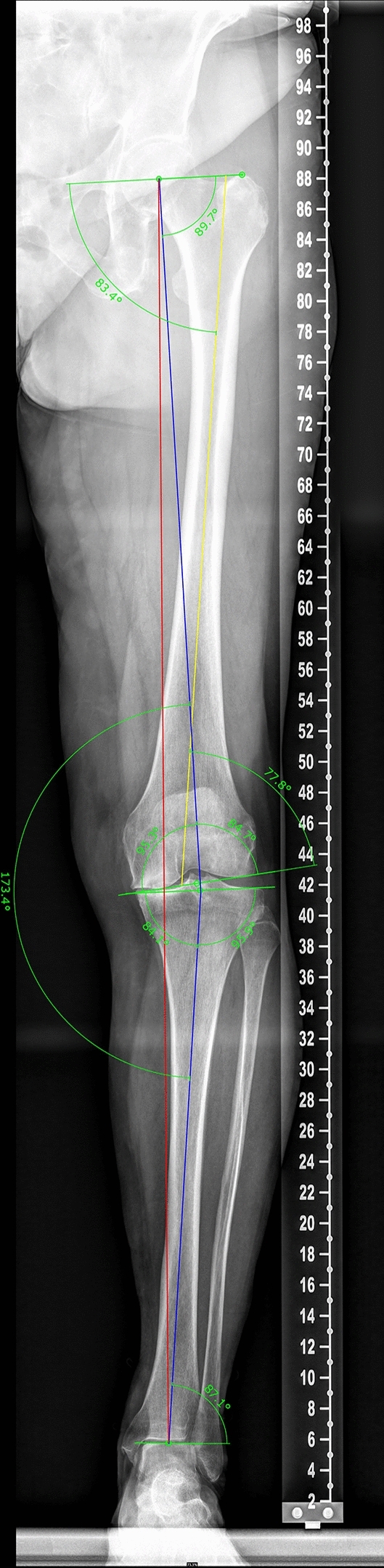
Schematic picture of the digital measurements performed on the AP radiograph of a total leg in the standing position

The joint lines, joint centers, and joint angles were marked on long leg radiographs and measured by hand according to Paley’s definition using mediCAD^®^ Version 6.0 (mediCAD Hectec GmbH, Germany). All axes were drawn in the frontal plane [19]. First, the joint orientation lines and then the anatomical and mechanical axes through the femur as well as the middiaphyseal line through the tibia were reconstructed. The Mikulicz Line was marked, and the total joint angles as well as the hip–knee–ankle angle (HKA) were measured (Fig. 2). All measurements were provided with one decimal number and performed twice by two independent investigators. Inter- and intraclass correlation analyses were calculated and revealed excellent agreement with values greater than 0.8.

The study protocol was approved by the local ethics committee (30-253 ex 17/18).

### Statistical analysis

Explorative and descriptive analyses were performed. According to Hirschmann et al. [9], limb phenotypes were classified based on the HKA. The varus limb phenotype was defined as HKA < 178.5°, the neutral phenotype as HKA between 178.5° and 181.5°, and the valgus phenotype as HKA larger than 181.5° [9]. In addition, the femoral phenotype based on the femoral mechanical angle [FMA, i.e., the mechanical medial distal femoral angle (mMDFA)] as well as the tibial phenotype based on the tibial mechanical angle [TMA, i.e., the medial proximal tibial angle (MPTA)] was calculated [9].

Statistical analyses were performed with Stata Version 16.1 for Mac (StataCorp, College Station, Texas, US). Means and medians were provided with corresponding standard deviations and interquartile ranges (IQRs). Pre- and postoperative alignment of the leg axes was compared with a paired t test. The change in leg axis alignment from the preoperative to the postoperative status was assessed with a chi-squared test. Differences in mechanical and anatomical angles depending on preoperative limb phenotype were performed with one-way analysis of variance (ANOVA) and post hoc pairwise comparisons of means.

Inter- and intraclass correlations were calculated for agreement between raters. A priori power analysis was calculated with respect to a magnitude of differences of 10% of change from varus to neutral or valgus to neutral with a power greater than 80%, and a *p* value of < 0.05 was considered to be statistically significant.

## Results

Seventy-one patients were female (63.4%), and 41 were male (36.6%). The mean patient age at surgery was 71.6 ± 8.4 years. According to the HKA by Hirschmann et al. [9], 67 patients had a preoperative varus alignment (59.8%), 36 had a valgus alignment (32.1%), and 9 had a neutral alignment (8.1%). Preoperatively, 29 patients (25.9%) had neutral femoral phenotypes according to Hirschmann et al., 30 had a valgus femoral phenotype (26.8%), and 53 had a varus femoral phenotype (47.3%). Regarding preoperative tibial phenotypes, 41 patients (36.6%) had neutral, 45 had valgus (40.2%), and 26 had varus phenotypes (23.2%).

Following TKA, 30 patients had a varus alignment (26.8%), 30 a valgus alignment (26.8%), and 52 a neutral alignment (46.4%; Table 1). Significantly more patients with preoperative varus alignment shifted to neutral alignment (*n* = 31; 46.3%) than did patients with preoperative valgus (*n* = 14; 38.9%; *p* < 0.001). Moreover, only 43.3% (*n* = 29) of patients with preoperative varus showed a persistent varus postoperatively, while 58.3% (*n* = 21) of patients with preoperative valgus still had a valgus alignment. Likewise, in the subgroup of males (*p* = 0.015) and females (*p* < 0.001), similar observations with regard to changes in the long leg axis depending on preoperative leg alignment were found. Changes in femoral and tibial phenotypes from pre- to postoperative status are depicted in Table 1.

**Table 1:** Pre- and postoperative alignment as defined by the hip-knee-ankle angle (HKA), FMA, and TMA according to Hirschmann et al. [9] (*n* = 112; *p* < 0.001)

HKA			
	Postoperative limb phenotype		
	Neutral	Varus	Valgus
Preoperative limb phenotype (*n*; %)			
Neutral	7 (77.8)	0 (0.0)	2 (22.2)
Varus	31 (46.3)	29 (43.3)	7 (10.4)
Valgus	14 (38.9)	1 (2.8)	21 (58.3)

FMA			
	Postoperative femoral phenotype		
	Neutral	Varus	Valgus
Preoperative femoral phenotype (*n*; %)			
Neutral	11 (37.9)	18 (62.1)	0 (0.0)
Varus	8 (15.1)	45 (84.9)	0 (0.0)
Valgus	10 (33.3)	16 (53.4)	4 (12.3)

TMA			
	Postoperative tibial phenotype		
	Neutral	Varus	Valgus
Preoperative tibial phenotype (*n*; %)			
Neutral	8 (19.5)	0 (0.0)	33 (80.5)
Varus	10 (38.5)	(0.0)	16 (61.5)
Valgus	5 (11.1)	(0.0)	40 (88.9)

### Mean axis deviation (MAD) and anatomical femorotibial angle (aTFA)

There was a significant overall difference in the pre- to postoperative change in MAD (*p* < 0.001) as well as aTFA (*p* < 0.001) depending on the preoperative leg alignment (Table 2). Specifically, the differences in mean change in MAD between pre- and postoperative were most notable in the valgus vs. varus group with − 11.2 ± 0.7 mm (*p* < 0.001), followed by the valgus vs. neutral group with − 5.8 ± 1.2 mm (*p* < 0.001). For varus vs. neutral, the difference was significant at 5.4 ± 1.1 mm (*p* < 0.001).

**Table 2:** Mean changes in measurements and angles from pre- to postoperative status depending on the preoperative leg alignment according to Hirschmann et al. [9]

Mean pre- to postoperative difference	Preoperative leg alignment			*p* value
	Neutral	Varus	Valgus	
MAD	0.7 ± 1.8 mm	6.0 ± 2.6 mm	− 5.9 ± 3.6 mm	**< 0.001**
aTFA	− 0.1° ± 2.5°	6.8° ± 4.1°	− 6.9° ± 5.6°	**< 0.001**
AMA	0.5° ± 0.5°	0.2° ± 0.6°	0.1° ± 0.8°	0.225
Femoral angles				
aMPFA	− 1.2° ± 1.7°	− 1.0° ± 3.6°	− 1.0° ± 4.0°	0.980
mMPFA	− 0.4° ± 1.7°	− 1.2° ± 4.5°	− 0.7° ± 3.9°	0.752
aMDFA/aLDFA	− 2.1° ± 7.9°	0.6° ± 5.2°	3.4° ± 4.9°	**0.008**
mMDFA/mLDFA	1.7° ± 2.3°	1.0° ± 3.3°	4.2° ± 3.0°	**< 0.001**
Tibial angles				
MPTA/LPTA	0.1° ± 1.6°	− 3.5° ± 2.9°	0.1° ± 2.7°	**< 0.001**
MDTA/LDTA	− 3.1° ± 2.6°	− 0.1° ± 4.5°	− 2.0° ± 3.6°	**0.022**

Significant *p*-values highlighted in bold

For the aTFA, the mean changes from pre- to postoperative were highest in the valgus vs. varus group (− 13.7 ± 0.9°; *p* < 0.001), followed by the valgus vs. neutral (− 6.9 ± 1.7°; *p* < 0.001) and varus vs. neutral groups (6.9 ± 1.6°; *p* < 0.001).

### Femoral angles

There was no significant overall difference in the anatomical medial proximal femur angle (aMPFA; *p* = 0.980; Table 2) or the mechanical medial proximal femur angle (mMPFA; *p* = 0.752; Table 2).

The mMDFA (*p* < 0.001) and anatomical distal femoral angle (aMDFA; *p* = 0.008) showed a highly significant overall difference in all four angles depending on the preoperative leg alignment (Table 2). In detail, a greater difference in aMDFA/anatomical lateral distal femur angle (aLDFA) was found for preoperative valgus vs. neutral alignment (5.5° ± 2.0°; *p* = 0.007) than for preoperative valgus vs. varus alignment (2.8° ± 1.1°; *p* = 0.013). The difference in preoperative varus vs. neutral alignment was not statistically significant (2.7° ± 1.9°; *p* = 0.160).

In addition, the mMDFA/mechanical lateral distal femur angle (mLDFA) changed significantly depending on the preoperative leg alignment (*p* < 0.001; Table 2). Specifically, significant changes in mMDFA/mLDFA were found for valgus vs. varus (3.2° ± 0.6°; *p* < 0.001) and valgus vs. neutral (2.4° ± 1.2°; *p* = 0.038) but not for varus vs. neutral preoperative leg alignment (− 0.8° ± 1.1°; *p* = 0.488). The aMDFA/aLDFA and mMDFA/mLDFA changed the most upon surgery in the case of preoperative valgus alignment.

### Tibial angles

There was a highly significant overall difference in all four tibial angles depending on the preoperative limb phenotype (Table 2). For the MPTA/lateral proximal tibia angle (LPTA), the greatest differences were found between preoperative valgus and varus alignment (3.6° ± 0.5°, *p* < 0.001) as well as varus and neutral alignment (− 3.6° ± 1.0°, *p* < 0.001). On the other hand, no significant difference between preoperative valgus and neutral leg alignment was present (0.1° ± 1.0°; *p* = 0.936).

As opposed to MPTA/LPTA with the largest overall difference from pre- to postoperative status seen in the preoperative varus phenotype, the greatest pre- to postoperative change in medial distal tibial tibia angle (MDTA)/lateral distal tibia angle (LDTA) was observed in preoperative neutral alignment (− 3.1° ± 2.6°; Table 2). There was a significant difference between varus and neutral alignment (3.0° ± 1.5°, *p* = 0.044) as well as between valgus and varus alignment (− 2.0° ± 0.9°, *p* = 0.023) but not between valgus and neutral alignment (1.0° ± 1.5°; *p* = 0.512).

### AMA (anatomical to mechanical angle)

No significant overall difference in AMA could be found from pre- to postoperative status depending on the preoperative leg alignment (*p* = 0.225; Table 2).

## Discussion

The aim of this study was to determine the amount of change in the long leg axis depending on the preoperative knee phenotype using the mechanically aligned extension-first, gap-balanced technique. The hypothesis was that the preoperative limb phenotype would have an impact on the extent of change in the postoperative leg axis.

We found that using an extension-first mechanically aligned surgical technique, varus phenotypes predominantly result in neutral leg axes or remain varus, neutral phenotypes remain neutral, and valgus phenotypes remain valgus or change to neutral phenotypes.

Varus deformities are very common deformities in patients with end-stage knee osteoarthritis [1]. The complexity lies in bony differences on one hand and a large variability of gap sizes from extension to flexion on the other hand [2, 4, 17]. With the extension-first technique considering both bony and soft tissue aspects, 46.3% of preoperative varus knees had a neutral alignment postoperatively. On the other hand, this was also true for 38.9% of preoperative valgus knees. In detail, 43.3% of varus knees persisted postoperatively, in line with a study by Heyse et al., proposing persisting varus malalignment in 30.2% of varus knee osteoarthritis patients [6]. In contrast with our results, Schiffner et al. reported a persisting varus deformity in 33 of 148 patients (22.3%), whereas the majority showed a neutral alignment postoperatively (*n* = 115; 77.7%) [25].

In comparison to the varus knee, a valgus knee is challenging due to its contracted lateral soft tissue structures [15]. A recent study reported that a residual valgus after TKA for valgus knee osteoarthritis might cause postoperative patella instability due to abnormal proportions of the patella tilt angle and the congruence angle, despite no significant effect on the short-term outcome [14]. In valgus knee alignment, the surgical approach may influence outcomes, with a lateral approach being associated with improved postoperative alignment and patellar tilt [5, 15, 18]. Nikolopoulos et al. showed that a residual valgus deviation occurred in 9% of cases when using a lateral parapatellar arthrotomy, as opposed to 32% when using a medial approach [18]. In the current study, 58.3% of preoperative valgus knees persisted in valgus alignment using the same medial approach in each case. In light of the studies mentioned above, the use of medial parapatellar arthrotomy might contribute to persisting valgus alignment. Correspondingly, only 38.9% of patients with preoperative valgus showed a neutral alignment postoperatively. On the other hand, 77.8% of preoperative neutral phenotypes persisted postoperatively, while 22.2% changed to a valgus alignment.

A 3D-CT study by Slevin et al. reported no correlation between coronal alignment and clinical outcomes [26], and Sappey-Marinier et al. presented a rate of only 60% successfully reconstructed neutral alignments in 1078 cases aimed at neutral mechanical alignment. This is in contrast to our findings (26.8% varus postoperatively), as they observed approximately persisting varus in 9% of cases postoperatively. Furthermore, neutral and valgus phenotypes persisted in 35% and 8% of patients, respectively, in contrast to the present study [24].

We want to underline the limitations that correlations of changes in leg axis alignment with clinical outcome were not evaluated and that the preoperative limb phenotype was assessed on X-rays already presenting osteoarthritis and on no X-rays taken in the second decade of the patients´ lives.

The benefits of this study are that all cases were operated on or under the direct supervision of one surgeon and that all measurements were taken twice by two observers with substantial inter- and intraclass agreement on X-rays, which are still considered the primary imaging technique [13].

## Conclusion

Using an extension-first mechanically aligned surgical technique, varus phenotypes predominantly result in neutral leg axes or remain in varus, neutral phenotypes remain neutral, and valgus phenotypes remain in valgus or change to neutral phenotypes. Preoperative valgus influenced this technique to a greater extent than estimated in previous investigations, and we believe that this should be considered in extension-first mechanically aligned TKA for patients with valgus knee osteoarthritis.
